# Pathophysiology, Clinical Manifestations and Diagnosis of Immune Thrombocytopenia: Contextualization from a Historical Perspective

**DOI:** 10.3390/hematolrep16020021

**Published:** 2024-04-03

**Authors:** Daniel Martínez-Carballeira, Ángel Bernardo, Alberto Caro, Inmaculada Soto, Laura Gutiérrez

**Affiliations:** 1 Department of Hematology, Hospital Universitario Central de Asturias (HUCA), 33011 Oviedo, Spain; angel.bernardo@sespa.es (Á.B.); alberto.caro@sespa.es (A.C.); inmaculada.soto@sespa.es (I.S.); 2 Platelet Research Lab, Instituto de Investigación Sanitaria del Principado de Asturias (ISPA), 33011 Oviedo, Spain; gutierrezglaura@uniovi.es; 3 Department of Medicine, University of Oviedo, 33006 Oviedo, Spain

**Keywords:** antiplatelet antibodies, clinical manifestations, diagnosis, history, immune thrombocytopenia (ITP), pathogenesis, platelets

## Abstract

Immune thrombocytopenia (ITP) is an autoimmune disease characterized by an isolated decrease in the platelet count and an increased risk of bleeding. The pathogenesis is complex, affecting multiple components of the immune system and causing both peripheral destruction of platelets and impaired central megakaryopoiesis and platelet production in the bone marrow. Here, we intend to contextualize the current knowledge on the pathophysiology, terminology, epidemiology, clinical manifestations, diagnosis, and prognosis of ITP from a historical perspective and the first references to the never-stopping garnering of knowledge about this entity. We highlight the necessity to better understand ITP in order to be able to provide ITP patients with personalized treatment options, improving disease prognosis and reducing the incidence or frequency of refractoriness.

## 1. History of Immune Thrombocytopenia and Platelets

Before platelets were discovered, the identification of immune thrombocytopenia (ITP) was based exclusively on the presence of purpura in an otherwise healthy individual. In medicine, purpura is a general term for reddish-purple skin lesions produced by bleeding in the dermis or subcutaneous tissues. It was recognized as a clinical sign as early as the Greco-Roman period by physicians such as Hippocrates and Galen, who described the condition as red “eminences” or spots associated with pestilential fevers [1]. In 1735, a German physician and poet named Paul Gottlieb Werlhof provided the first detailed description of what we know today as ITP, separating it from the rest of the purpuras and naming it “*morbus maculosus hemorrhagicus*”. He described the case of a 16-year-old young woman with cutaneous hemorrhagic symptoms, epistaxis, and gingival bleeding [2]. From then on, it became known as “purpura” or “Werlhof’s disease”, at a time when platelets and other components of the blood were completely unknown.

Platelets were first referred to in 1841, when the English physician, Willian Addison, described them as “*extremely minute*…*granules*” in the blood. He considered these particles as granules from ruptured leukocytes, and they were not yet seen as independent cellular elements [3,4]. Max Schultze published the first accurate and convincing description of platelets as part of a study mainly devoted to white blood cells in 1865. He studied the different types of leukocytes, but also identified other corpuscles that he named “*spherules*”: “*Because of their pallor and very small size, which is 6–8 times smaller than the red cells, the individual spherules can only be recognized with good strong lens*” [5]. The concept of Schultze’s corpuscles as “dust of the blood” originating from leucocytes, mainly during infectious diseases, was supported by many other contemporary hematologists [1]. The Italian pathologist Bizzozero introduced the term “*platelets*” in 1882. He called the granules “*piastrine*” in Italian, “*petite plaques*” later “*plaquettes*” in French, and “*Blutplättchen*” in German. In addition to making a detailed description of their morphology as discoid corpuscles without a nucleus, he was the first to describe platelets as a third morphological element of the blood, unrelated to erythrocytes and leukocytes, thus contradicting his peers. He was also the first to demonstrate the role of platelets in hemostasis and their contribution to thrombosis; however, he could not demonstrate their origin [5,6,7]. One year later, in 1883, Brohm identified the association between thrombocytopenia and the syndrome described by Werlhof [8] and so did Denys, independently, years later in 1887 [9]. The first platelet count documenting thrombocytopenia in this purpura was performed by Hayem in 1889 [10]. Overall, it took 150 years to find an explanation for the “*morbus maculosus haemorrhagicus*”. In 1890, William Henry Howell described and named megakaryocytes (“*I shall speak of them hereafter as megakaryocytes, or large nucleated giant cells…*”) [11]. In 1906, J. H. Wright described platelets as fragments of the cytoplasm of megakaryocytes. In this way, the basic elements of megakaryopoiesis were established [12,13]. From then on, efforts would be directed mainly at discovering the cause of thrombocytopenia in Werlhof’s disease.

## 2. Pathophysiology of ITP

In 1915, Ernest Frank proposed that the cause of thrombocytopenia was the suppression of megakaryocytes by a toxic substance produced in the spleen [14]. A year later (1916), a Viennese medical student named Paul Kaznelson proposed that the cause of thrombocytopenia in ITP was an increased destruction of platelets in the spleen. He also reported the first case of splenectomy with a good response: a woman with a history consistent with chronic ITP [15]. It was no less than 30 years later, in 1946, when Dameshek and Miller studied the bone marrow from patients with ITP and found an increase in the total number of megakaryocytes, while most of those cells did not produce platelets. Effective platelet production appeared to increase after splenectomy [16,17].

The debate on the mechanisms of thrombocytopenia in ITP, peripheral destruction of platelets versus altered production, was apparently resolved in 1951. The immune-mediated destruction of platelets was demonstrated thanks to the experiment carried out by William J. Harrington and colleagues. Harrington observed a child with purpura born to a mother with chronic ITP, a condition already described by Dohrn in 1873. The child’s purpura resolved spontaneously within 3 weeks while the mother remained thrombocytopenic. He then suspected that there had been a transfer of a “humoral factor” from the mother to the baby [1,18]. To confirm his suspicion, Harrington transfused himself with blood from an ITP patient. Harrington’s platelet count, previously normal, dropped sharply and returned to normal over the following days. The same experiment was subsequently carried out with healthy volunteers with the same result. He concluded that the platelet clearance in ITP was due to a “humoral factor” [19]. That same year, Evans et al. described the association of positive Coombs hemolytic anemia with ITP, suggesting an autoimmune mechanism and an antiplatelet antibody as the cause of thrombocytopenia [20].

### 2.1. Autoantibodies

In 1965, Shulman et al. observed that the humoral factor causing thrombocytopenia was Immunoglobulin G (IgG) [21]. In 1982, Van Leeuwen et al. found that antibodies from patients with ITP bound to normal platelets, but not to platelets from patients with Glanzmann’s thrombasthenia; thus, he speculated that patients with ITP produced autoantibodies against platelet glycoprotein (GP) IIb or GPIIIa since patients with thrombasthenia lack either of these proteins [22]. Two years later, Woods et al. demonstrated the presence of autoantibodies against GPIIb/IIIa and GPIb in ITP patients [23,24]. Subsequently, two new methods were described for the first time to identify these autoantibodies. These techniques, the Monoclonal Antibody-specific Immobilization of Platelet Antigen (MAIPA) and the immunobead assay [25,26], were capable of detecting the presence of either free antibodies in serum or those bound to platelets, with a sensitivity of 49–66% and a specificity of 78–92% [27]. It was thus confirmed that the humoral factor described by Harrington and colleagues was an antibody directed against the glycoproteins of the platelet membrane.

Almost in parallel to the discovery of platelet autoantibodies, bone marrow damage as a cause of thrombocytopenia returned to prominence in the 1980s, as it was shown that antibodies against platelet antigens also bind to megakaryocytes [28,29,30]. Two much more recent studies (2003 and 2004) supported the hypothesis that ITP autoantibodies suppress the production and maturation of megakaryocytes [31,32]. Other investigations also confirmed the role of megakaryocytes in the pathogenesis of the disease by showing extensive megakaryocyte abnormalities suggestive of a “non-classical apoptosis” mechanism in ITP patients [33]. Thus, the dogma that ITP was a disorder due exclusively to the accelerated destruction of platelets was effectively challenged.

### 2.2. T Lymphocytes

New evidence suggested that the pathogenesis of ITP was more complex than anticipated; in 1991, Semple et al. demonstrated that the T-cell compartment was also involved in the disease. Specifically, they showed that the disease could result from an abnormal defect in T helper (Th) cells, which could direct autoreactive B cells to produce autoantibodies [34,35,36]. Subsequent studies showed that regulatory T cells (Tregs) also contributed to disease progression through a loss of tolerance [37,38]. In 2003, Olson et al. found that cytotoxic T cells (Tc) in ITP patients deprived of platelet antibodies, could also cause thrombocytopenia via direct lysis [39]. A 2007 study suggested that CD8+ T cells lead to impaired platelet production by suppressing megakaryocyte apoptosis [40]. Summarizing, ITP is a disease caused by the dysfunction of the immune system at multiple levels, that affects multiple arms and players in the platelet–megakaryocytic lineage and its homeostasis.

### 2.3. Thrombopoietin

Kelemen was the first to coin the term “thrombopoietin” in 1958 to describe a humoral substance responsible for the recovery of the platelet count after thrombocytopenia [41]. Thrombopoietin (TPO) is a protein synthesized mainly in the liver and the main regulatory hormone of megakaryopoiesis. However, it was not until 1994 that its purification and cloning were achieved [42,43,44]. TPO acts through the TPO receptor (also known as MPL) promoting the proliferation, differentiation, and maturation of megakaryocytes and precursors. Subsequently, it was observed that TPO levels in patients with ITP were low compared to those with thrombocytopenia due to aplastic anemia, suggesting inappropriate stimulation of thrombopoiesis in ITP [45,46].

Today, we know that TPO levels are inversely proportional to the number of platelets or, to be more exact, to the megakaryocytic–platelet mass, which therefore includes elements in the marrow and in the circulation. Subjects with normal platelet counts have TPO levels in the normal physiologic range, while patients with central thrombocytopenia have high circulating levels. Paradoxically, TPO levels in patients with ITP are lower than expected for the degree of thrombocytopenia. We now know that this could be explained by the rapid splenic clearance of both platelets and TPO bound to its platelet MPL receptor, thus causing a decrease in circulating TPO levels [47]. However, the contribution of the increased megakaryocyte mass in the bone marrow in this apparent imbalance cannot be ruled out.

The regulatory mechanisms of TPO production have been the subject of discussion for decades. In addition to the “autoregulation” model of TPO discussed in the previous paragraph, an additional new model has recently been described in which the loss of sialic acid, characteristic of aging circulating platelets, determines their clearance. Desialylated (senescent) platelets are removed from the circulation in hepatocytes via the Ashwell–Morell receptor (AMR). The binding of platelets to this receptor results in a signaling cascade where JAK2 and STAT3 participate, inducing the production of TPO messenger RNA, thus stimulating its hepatic synthesis. In this way, the replacement of “aging” by “young” platelets in the circulation is guaranteed; “aging” platelets themselves stimulate TPO production, thus stimulating their production in the bone marrow. In addition to TPO, various inflammatory factors have also been postulated as regulators of megakaryopoiesis, such as IL-6, among others [48,49].

To delve deeper into the history of ITP, we recommend the reader to peruse the more extensive and detailed Stasi and Newland review [1]. Table 1 summarizes the major referenced historical advances in ITP knowledge.

### 2.4. Current Pathophysiological Model in ITP

The pathogenesis of the disease remains unknown, because it most likely involves both genetic and acquired factors. A trigger acts on a genetic susceptibility, often unknown, such as infections or situations that affect the immune system (neoplasms, autoimmune diseases, vaccines…). Harrington’s experiment marked the beginning of the immunological era of ITP, focusing its etiology on antibody-mediated platelet destruction. However, we currently know that its pathophysiology is much more complex, affecting multiple components of the immune system that mediate both the peripheral destruction of and inadequate production of platelets [50].

In ITP, immunoglobulin G-type autoantibodies are generally the most frequent (IgA and IgM are less frequent). These autoantibodies are produced by autoreactive B lymphocytes and bind to glycoproteins of the platelet membrane, which favors the clearance of platelets from the circulation through the reticuloendothelial system (SRE) of the liver and spleen. The main platelet targets are GPIIb/IIIa and GPIb/IX, although GPIa/IIa, GPIV, and GPV may also be targeted [50]. The cytokine, BAFF (B cell activating factor), is increased, which stimulates autoreactive B cells, thus boosting antiplatelet antibody production. Various mechanisms have been suggested to be responsible for the production of auto-antibodies in ITP, such as antigenic cross-reactivity (mimicry), somatic mutations, or defects in the elimination of autoreactive clonal B cells [51]. The current and prevailing model of platelet clearance in ITP postulates that the platelet target of autoantibodies determines the pathway of clearance. The anti-GPIIb/IIIa antibody-mediated destruction of platelets occurs primarily in the spleen where the interaction between the Fc fraction of platelet-associated antibodies and the Fc receptors of macrophages initiates phagocytosis. However, anti-GPIb antibodies trigger the loss of sialic acid in platelets, and this process diverts platelet clearance towards the liver through binding to its AMR receptors (Fc-independent mechanism). The process by which the binding of anti-GPIb antibodies induces platelet desialylation seems to be caused by platelet basal activation, followed by degranulation and translocation of neuraminidase to the membrane, which removes the sialic groups from glycoproteins [52,53]. Knowledge of this modality of clearance through AMR provides a potential explanation for the refractoriness to splenectomy, as well as steroid and intravenous immunoglobulin therapies in a certain percentage of patients [49]. Furthermore, platelet autoantibodies also induce platelet apoptosis and allow complement fixation, favoring their destruction [54,55].

On many occasions (30–40% of cases), the antibodies are not detected, which also suggests the participation of other alternative immunological mechanisms, such as cellular immunity and an imbalance of regulatory mechanisms, with the participation of cytotoxic and regulatory T lymphocytes [56,57].

In ITP, there is a loss of immunological tolerance to specific self-antigens. There is an imbalance in the Th1/Th2 lymphocyte ratio, resulting in the prevailing proinflammatory effect (Th1) with the release of cytokines that activate the immune system (IL-2, TNF-α, TNF-β, and IFN-γ) and a decrease in anti-inflammatory cytokines produced by Th2 cells (IL-4, IL-5, IL-6, IL-10, and IL-13) [50,58]. Treg lymphocytes are a subpopulation of T cells specialized in immune suppression and contribute to the maintenance of immune tolerance; their dysfunction determines the pathogenesis of several autoimmune diseases. Different studies have shown a decrease in/dysfunction of Treg lymphocytes in patients with ITP when compared to healthy subjects. Th17 lymphocytes are a subgroup of Th lymphocytes that modulate the proinflammatory response by producing IL-17, and play a role opposite to that of Treg lymphocytes. Several studies have shown an increase in Th17 lymphocytes and the proinflammatory cytokine IL-17 in autoimmune diseases and in active ITP. Decreased and defective regulatory B cells (Bregs) have also been described in ITP, with deficient production of the anti-inflammatory cytokine IL-10 [50]. Additionally, platelet destruction in ITP patients has been documented to also be mediated by CD8+ cytotoxic T lymphocytes, which are activated and capable of directly lysing platelets [56]. IL-21 is synthesized by helper T cells and is increased in ITP patients compared to healthy controls. Higher IL-21 levels could be predictors of relapse in ITP, as it has been proposed [59].

**Figure 1:**
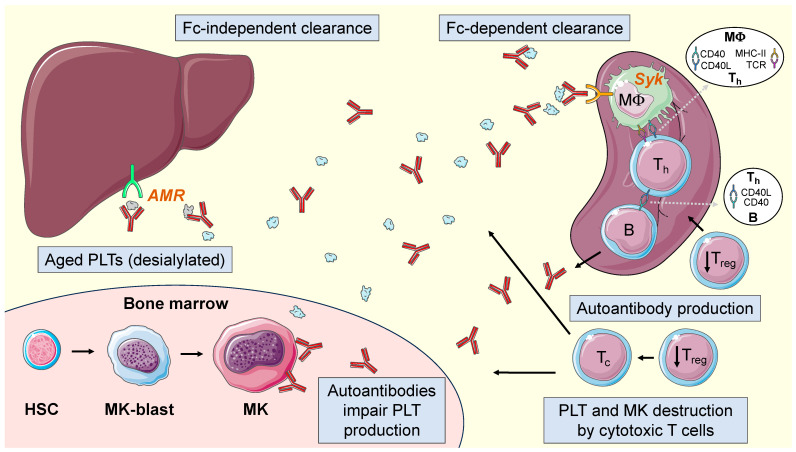
Pathophysiology of ITP. Dysfunction of regulatory T cells (T_reg_) leads to a disruption in the regulation of T helper cell (T_h_)-mediated B cell activation. B cells, in turn, produce autoantibodies in abundance that lead to opsonization, phagocytosis (by macrophages -MΦ-, and mediated by the spleen tyrosine kinase -Syk-) and complement activation, desialylation, and finally destruction of platelets. Autoantibodies further hinder megakaryocyte maturation, and autoreactive cytotoxic T cells (T_c_) destroy megakaryocytes and platelets. HSC, hematopoietic stem cell; MK-blast, megakaryoblast; MK, megakaryocyte; PLT, platelet; AMR, Ashwell–Morell receptor. The figure was generated using Servier Medical Art, provided by Servier, licensed under a Creative Commons Attribution 3.0 unported license (https://creativecommons.org/licenses/by/3.0/). Image adapted from: Singh, A.; et al. *J. Clin. Med*. **2021,** *10 (4)*: 789. [60].

Platelet production in patients with ITP is also decreased. On the one hand, the strikingly low levels of TPO, compared to other central thrombocytopenias, contribute to this central defect. Although it seems that in patients with ITP there is an increase in the megakaryocytic mass in the marrow, it has been proven that this does not occur in all patients, and furthermore, it is not an increase that justifies the loss of the inversed balance between TPO levels and the number of platelets (or platelet/megakaryocyte mass). Moreover, megakaryocytes are also targeted by the same immune mechanisms that lead to peripheral platelet destruction. Megakaryocytes express GPIb and GPII/IIIa on their membrane, so they are also susceptible to becoming targets for autoantibodies; as it has been shown, autoantibodies induce morphological and physiological changes on megakaryocytes that lead to defective megakaryopoiesis. Likewise, autoreactive Tc lymphocytes accumulate in the bone marrow, thus causing the destruction of megakaryocytes via direct lysis. In fact, megakaryocytes in patients with ITP present various alterations such as a reduction in granules, vacuolization of the cytoplasm, smoothing of the plasma membrane, and a decrease in ploidy [50,51,56]. Figure 1 shows the different mechanisms involved in the pathogenesis of ITP.

## 3. Terminology

In 2008, a systematic review of the literature showed a lack of consensus regarding the terminology used for this disease. The authors of the review concluded that the terminology was confusing, and the heterogeneity in definitions was unacceptable in order to be able to make decisions, compare different studies, and share data; there was an urgent need for the standardization of the relevant terminology [61].

The International Working Group (IWG), after the meeting held in Vicenza in 2007, proposed a new standardized terminology, which was documented in 2009. The expert panel decided to avoid the term “idiopathic”, preferring the term “immune”, to emphasize the mechanism of the disease and chose “primary” to indicate the absence of an identifiable underlying cause. The term “purpura” was considered inappropriate, since hemorrhagic symptoms are absent or minimal in a large proportion of cases. The acronym ITP was retained due to its widespread use and its usefulness for literature searches. In this way, “ITP” came to mean immune thrombocytopenia, instead of idiopathic thrombocytopenic purpura, as it was classically known. In addition, a platelet count of less than 100 × 10^9^/L was established as the clinical threshold for diagnosis. It was considered that a predefined uniform limit is more convenient for practical use and for comparing studies. The threshold of 150 × 10^9^/L platelets, previously used by the majority, was not appropriate since a non-negligible percentage of the healthy population and pregnant women have platelet numbers of between 100 and 150 × 10^9^/L without constituting any pathology. Therefore, primary ITP was defined as isolated thrombocytopenia < 100 × 10^9^/L in the absence of other causes that may justify it. The term “secondary immune thrombocytopenia” or “secondary ITP” has been proposed to include all forms of immune thrombocytopenia except primary ITP. In these cases, the acronym “ITP” should be followed by the name of the associated disease. “Severe ITP” refers to ITP that presents at diagnosis with bleeding symptoms that require treatment, or when new bleeding symptoms require additional therapeutic intervention with a different platelet-enhancing agent or an increased dose. “Refractory ITP” refers to ITP that does not respond or relapses after splenectomy and is severe (associated with bleeding or risk of bleeding that requires therapy) [62]. Since splenectomy is becoming a rare procedure today, this definition is outdated. In fact, refractoriness is now commonly applied to ITP patients that do not respond to multiple lines of treatment, independently of splenectomy.

The phases of the disease were also defined. Traditionally, ITP was differentiated into “acute” and “chronic” if the evolution of the disease was less than or more than 6 months. According to the new terminology, ITP is called newly diagnosed, persistent, and chronic according to the time of evolution of less than 3 months, between 3 and 12 months, and more than 12 months, respectively. Regarding the response criteria, they were defined as follows: Complete response (CR), platelet count ≥ 100 × 10^9^/L and absence of bleeding; Response (R), platelet count ≥ 30 × 10^9^/L and <100 × 10^9^/L, an increase of more than twice the baseline number and absence of bleeding; Non-response (NR), platelet count < 30 × 10^9^/L or an increase of less than twice the baseline value or bleeding. The expert panel decided to avoid the frequently used term “partial response” or “minimal response”, as it was confusing, as there was heterogeneity in the criteria used [62]. As we see, all these definitions are governed by the time of evolution of the disease, clinical criteria, or therapeutic response, but we still lack a pathophysiological classification that allows personalized treatment.

## 4. Epidemiology

ITP is a rare disease with an annual incidence that varies from 1.1 to 12.5/100,000 inhabitants/year [63], being similar between children and adults. The distribution is trimodal with three peaks: one in children between 1 and 5 years old, another in young adults (clear female predominance at this peak), and another in adults > 60 years old. Overall, the incidence is slightly higher in women [64,65]. According to a nationwide population-based study in France, the natural history of pediatric ITP is different from that of adults since only 35.7% of children with primary ITP will progress to persistent or chronic disease, while adult primary ITP will become persistent or chronic in 66.7% of the cases [65]. Of note is that this study included patients aged 13–17 years old as pediatric patients. Therefore, given that chronification is more common in adults, the prevalence is higher in them, being 4.6 per 100,000 inhabitants of pediatric age and 9.5–23.6 per 100,000 inhabitants of adult age [66,67].

## 5. Clinical Manifestations

ITP is a disorder of primary hemostasis, and therefore the predominant symptoms are skin and mucosal bleeding. In the Spanish TIMES registry, 484 patients were included, both children (12.4%) and adults (87.6%), with primary (n = 433) and secondary (n = 51) ITP. Clinical manifestations at diagnosis were observed in 72% of patients with primary ITP and 67% of patients with secondary ITP, mainly cutaneous (61 and 63%, respectively) and oral cavity bleedings (29% and 24%), and epistaxis (17% and 24%). Potentially severe bleedings were rare (gastrointestinal bleedings 4% and 14%; intracranial bleedings 1% and 2%). Other bleeding symptoms included muscle hematoma (6% and 0%), metrorrhagia (5% and 4%), and hematuria (2% and 6%) [68].

A retrospective analysis carried out in 2010 showed the first evidence suggesting a prothrombotic state in ITP, since it observed an increase in venous thrombosis compared to controls without ITP, without significant differences in terms of arterial thrombosis [69]. Subsequent studies have confirmed this observation, thus demonstrating that patients with primary ITP have a higher risk, not only of venous, but also arterial thrombosis compared to the general population and regardless of the platelet count [70,71,72,73,74,75]. The risk factors for the development of thrombosis in ITP can be divided into three groups: related to the patient (classic risk factors such as immobility, surgery, cancer, tobacco…), related to the treatment (splenectomy, intravenous immunoglobulins [IVIG], corticosteroids, and TPO receptor agonists [TPO-RA]), and related to ITP. Among the factors intrinsic to the disease itself, several have been postulated: increase in the fraction of immature and hyperactive platelets, increase in proinflammatory cytokines, presence of circulating megakaryocytes… [76]. A study has observed that, compared to healthy subjects, patients with ITP present an increase in microparticles, an increase in resistance to protein C, and clot formation that is more resistant to fibrinolysis due to elevated plasma levels of PAI-1 [77]. These series of compensatory mechanisms constitute a procoagulant profile that would be responsible for less frequent and severe hemorrhagic symptoms in patients with ITP when compared to patients with thrombocytopenia of central origin and the same platelet count.

Patients with ITP also have an increased risk of infections that have classically been linked to immunosuppressive treatments or splenectomy. We now know that platelets play an important role in immunity and inflammation. In this sense, a study showed that the reduction in the number of platelets in ITP, regardless of treatment with corticosteroids, could increase the risk of infections [78].

Fatigue is a common symptom (22–39% of patients) with a great impact on the quality of life, although it is rarely perceived as a manifestation of ITP by hematologists [79]. The cause is not completely known, but it is probably multifactorial, with the presence of proinflammatory cytokines playing an important role, and anemia and iron deficiency, among others, possibly contributing. Both fatigue and other aspects of the quality of life should be considered when indicating therapy targeted at ITP as they could improve with treatment [80].

## 6. Diagnosis

Nowadays, the diagnosis of ITP continues to be one of exclusion, with no targeted, sensitive, and specific tests yet available [81,82]. There is no general agreement on the set of tests to be performed in the diagnostic process, and practice varies greatly. The evaluations to be performed in suspected ITP, according to our opinion and our clinical practice, are shown in Table 2.

The mandatory evaluations should be performed in all patients. It is essential to investigate a family history of thrombocytopenia and take a rigorous personal history (comorbidities, bleeding history, chronic medication, recent vaccinations, recent intake of drugs or herbal products, duration of thrombocytopenia…). The physical examination focuses on the search for hemorrhagic signs, hepatosplenomegaly, and lymphadenopathy. The peripheral blood smear is mandatory and should be the first step in the study of all thrombocytopenias in order to rule out pseudothrombocytopenia or abnormalities that indicate other associated pathologies. Platelets in patients with ITP are usually large and well granulated, accompanied by a high mean platelet volume and immature fraction. Despite its importance, the peripheral blood examination is the main reason for deviations in clinical practice in our country, as it has been reported that 22.8% of adult patients with primary ITP lack such simple cytological test [83]. Although some authors recommend the determination of blood group and Rh, it is not essential in our area, because it lacks diagnostic and therapeutic utility, since anti-D immunoglobulin is not part of the therapeutic options in Europe.

The prevalence of antiphospholipid antibodies (APLAs) in patients with primary ITP is higher compared to the general population and is estimated to be between 25 and 75% depending on the series [84]. They do not appear to affect the response to treatment and international guidelines do not recommend their routine determination in the absence of symptoms of antiphospholipid syndrome (e.g., thrombosis or history of fetal loss) [85]. Our recommendation is to perform a determination routinely at diagnosis since it emphasizes the autoimmune nature of thrombocytopenia and helps to profile the patient’s thrombotic risk.

Antinuclear antibodies (ANA) are present in 13–65% of adults with ITP and could be a predictor of chronicity [86] in addition to being useful to exclude systemic lupus erythematosus.

Regardless of age, routine bone marrow testing is not necessary for the diagnosis of ITP, as long as the clinical history, physical examination, complete blood count, and peripheral blood smear do not show abnormalities different from those observed in isolated thrombocytopenia. Bone marrow examination is appropriate in cases of relapse after remission, non-response to initial treatment, prior to splenectomy, or when other alterations are observed in the blood count or peripheral blood smear. If performed, it should include aspiration, biopsy and flow cytometry, and cytogenetic and molecular biology studies [85].

*Helicobacter pylori* (*H. pylori*) bacterium colonizes the stomach and gastrointestinal tract, infecting nearly 50% of the worldwide population. It is endemic in most countries of the world, with a reported prevalence of approximately 90% in Asia and the Mediterranean basin, and approximately 60% in Western Europe and North America. *H. pylori* infection is more prevalent in adults than children because of increased exposure with age [87]. Since a first study published in 1998 [88], different works have revealed a pathophysiological link between ITP and *H. pylori* infection. Interestingly, in many countries with a high prevalence of infection, bacterial eradication reverses thrombocytopenia in approximately 50% of ITP cases [89]. In Spain, the only study carried out in this sense shows a platelet response rate of 13% after the eradication of *H. pylori*, which is very low compared to the rest of the published series [90]. International guidelines recommend that detection of *H. pylori* with a breath or stool antigen test should be considered in adults with typical ITP, in those with digestive symptoms, and in those who come from high-prevalence areas [85].

A systematic review and meta-analysis of platelet auto-antibody tests (anti-GPIIb/IIIa or anti-GPIb) in the diagnosis of ITP showed that the sensitivity and specificity of direct tests were 53% and 93%, respectively. For indirect tests, sensitivity and specificity were 18% and 96%, respectively. Therefore, the determination of platelet autoantibodies in patients with ITP has high specificity, but low sensitivity. A positive test could be useful to confirm ITP, but a negative test does not rule it out [91]. Currently, international guidelines do not recommend the determination of platelet auto-antibodies [85].

### Differential Diagnosis

The differential diagnosis of ITP must be made along with the rest of the causes of thrombocytopenia, both secondary immune and non-immune (Table 3).

The differentiation between primary and secondary ITP is clinically relevant since the treatment of the underlying cause is essential in the latter. Experts have estimated, based on their clinical experience, that 20% of ITPs are secondary [92] and their frequency increases with age. Specifically, in a nationwide French study, 18% of ITPs diagnosed in adults were secondary. The main cause was hematological malignancy, followed by systemic autoimmune diseases, chronic viral infection, and primary immunodeficiency. In children, ITP was secondary in 2.4%, and the main causes were, in descending order, as follows: primary immunodeficiency, systemic autoimmune diseases, and hematological malignancies [65]. We must also highlight the importance of including drug-induced immune thrombocytopenia in the differential diagnosis, which can be triggered by a wide variety of medications, usually has an acute course and usually remits with withdrawal of the drug.

Given the absence of sophisticated and targeted tests, the differential diagnosis of ITP is often a challenge. A response to first-line therapy supports the diagnosis of ITP, while no response does not exclude ITP but increases the likelihood of other causes of thrombocytopenia. A real-life retrospective analysis showed a diagnostic error in 15% of adult patients diagnosed with primary ITP, as they ultimately turned out to have another cause of thrombocytopenia, either secondary immune or non-immune. The most common alternative diagnoses were myelodysplastic syndromes, familial thrombocytopenias, splenomegaly/hypersplenism, liver disease, and pseudothrombocytopenia [93]. In a series of 181 women with hereditary thrombocytopenias, 31% of them were initially labeled as ITP, having received, in many cases, unnecessary treatments such as immunosuppressants and splenectomy [94].

## 7. Prognosis

The natural history of ITP is different in pediatric and adult populations, since adults are more likely to develop chronic ITP. In general, up to almost 60% of adults end up developing chronic disease [64,95], while only 11% of children continue to manifest ITP a year after diagnosis [96]. In the pediatric population, a systematic review and meta-analysis identified the following predictors of chronicity: female sex, age ≥ 11 years, absence of infection or vaccination prior to diagnosis, insidious onset, platelet count ≥ 20 × 10^9^/L at the baseline, absence of mucosal bleeding, and the presence of antinuclear antibodies [97]. In adults, in the CARMEN registry, only the presence of ANAs at a titer ≥ 1/160 was associated with chronicity in the multivariate analysis [95]. However, in adults, this finding is still under debate since there are studies that did not find this association [64].

The overall mortality of patients with ITP is slightly higher than that of the general population (1.3–2.2 times higher according to different studies). In a Danish study published in 2014, with 20 years of follow-up, the overall mortality was higher than that of the general population, HR 1.5 [95% CI: 1.2–1.8], mainly due to an increase in mortality related to cardiovascular diseases, infections, hemorrhages, and hematological malignancies. However, there was no increase in mortality related to solid tumors [98]. We lack more up-to-date studies that reflect the lower use of corticosteroids or the impact of TPO-RA; therefore, we do not know exactly the mortality of patients with ITP in the current TPO-RA era.

Diabetes, kidney failure, vascular disease (arterial or venous thrombosis), and thyroid disease appear to have a higher incidence among patients with ITP. Likewise, there is also a higher incidence of hematological neoplasms such as lymphomas and leukemias, although the rates of these neoplasms may be overestimated due to errors in the diagnosis of ITP [71,99].

The prognosis of ITP worsens if it is secondary. One study that included patients with primary and secondary ITP found that those who were reclassified as secondary ITP within a median follow-up of 9.4 years had a mortality six times higher than the general population; however, the mortality of patients with primary ITP was only 1.3 times higher [100].

## 8. Areas for Future Work and Direction

ITP is an organ-specific autoimmune disease characterized by an isolated decrease in platelet counts and an increased risk of bleeding. The pathogenesis is complex, affecting multiple components of the immune system and causing both peripheral destruction of platelets and inadequate production in the bone marrow. There are not many publications that provide a historical view of ITP, contextualizing and updating, at the same time, on the pathophysiology, clinical aspects, and diagnosis of the disease.

Since Werlhof’s original description, we have learned a lot about the pathophysiology and management of ITP; however, important questions and challenges in regard to ITP remain to be resolved. Although more and more progress is being made in its pathogenesis, it is still not completely understood and is probably different in each patient. Basic pathophysiological aspects are still unknown, such as the functional capacity of platelets or cellular differentiation mechanisms. The diagnosis continues to be one of exclusion, without having sufficiently sensitive and specific tests as of today. This results in a risk of diagnostic errors and unnecessary treatments. There are also no laboratory tests that allow us to distinguish the predominant pathogenic pathway involved in each patient’s disease, which translates into a homogeneous treatment for all cases. We also lack biomarkers that allow us to recognize which patients will respond to a certain treatment, thus improving the efficiency of the therapeutic strategies and avoiding or reducing unnecessary treatments. Furthermore, ITP should be carefully studied within the different age groups, which should be defined by their particular pathophysiological manifestations. In particular, adolescents and young adults (AYAS; although there is no international consensus, the lower limit might reach 12 years while the upper limit might reach 40 years) have been misleadingly considered either as children or as adults, which could have an impact on the results described in previous reports, regarding prevalence and prognosis [65,96,97]. Clearly, ITP manifests with age-dependent features that, once acknowledged, will condition the personalized treatment of patients [101].

Thus, ITP continues to present new challenges for health professionals, aimed at improving the quality of life of patients. The future challenge should be the search for direct diagnostic methods, specific biomarkers that predict response, and the personalization of treatment. Basic research and clinical studies must go hand in hand to improve the management of patients with ITP.

## Figures and Tables

**Table 1:** ITP historical summary.

Year	Author	Discovery
1735	Werlhof [2]	“*Morbus maculosus hemorrhagicus*” (ITP) identified
1841	Addison [3]	The first-time platelets were referred to (“*extremely minute…granules*”)
1865	Schultze [5]	First accurate description of platelets
1882	Bizzozero [6]	Introduced the name “*platelets*” Role of platelets in hemostasis
1883	Brohm [8]	Association between thrombocytopenia and the Werlhof syndrome
1889	Hayem [10]	First platelet count documenting thrombocytopenia in this purpura
1890	Howell [11]	Description of megakaryocytes
1906	Wright [12,13]	Platelets as fragments of the cytoplasm of megakaryocytes
1915	Frank [14]	A toxic substance produced by the spleen causes thrombocytopenia
1916	Kaznelson [15]	Increased platelet destruction in the spleen First splenectomy
1946	Dameshek and Miller [16]	Increase in the total number of megakaryocytes in the bone marrow. Most of these cells did not produce platelets
1951	Harrington et al. [19]	“Humoral factor” (Immune etiology)
1951	Evans et al. [20]	Anti-platelet autoantibodies
1958	Kelemen et al. [41]	The first to coin the term “thrombopoietin”
1965	Shulman et al. [21]	The humoral factor causing thrombocytopenia is an immunoglobulin G
1978	McMillan et al. [28]	Anti-megakaryocyte autoantibodies
1982	Van Leeuwen et al. [22]	Anti-glycoprotein autoantibodies
1991	Semple et al. [34]	T cell abnormalities
1994	Bartley et al. [42] Kuter et al. [43] Lok et al. [44]	Purification and cloning of thrombopoietin
1996	Emmons et al. [45] Kosugi et al. [46]	TPO levels in ITP are low compared to central thrombocytopenia
2003	Chang et al. [31]	Anti-megakaryocyte autoantibodies
2004	McMillan et al. [32]	Anti-megakaryocyte autoantibodies
2004	Houwezijl et al. [33]	Megakaryocytic abnormalities
2015	Grozovsky et al. [48,49]	Desialylated platelets are removed from the circulation in hepatocytes via AMR

Abbreviation: AMR, Ashwell–Morell receptor; TPO, thrombopoietin.

**Table 2:** Recommendations for the study of patients with suspected ITP.

**Mandatory**
Patient history, family history, physical examination, CBC and reticulocytes, peripheral blood smear, basic coagulation test, liver function, folic acid and vitamin B12, immunoglobulin levels, serology (HIV, HBV, HCV)
**Recommended**
Anti-phospholipid antibodies, anti-thyroid antibodies and thyroid function, antinuclear antibodies, serum proteinogram
**Tests of potential utility**
Anti-glycoprotein antibodies, reticulated/immature platelet fraction, *H. pylori*, bone marrow examination (in selected patients), Viral PCR for EBV, CMV, and parvovirus, blood group and Rh, direct antiglobulin test, pregnancy test
**Tests of unproven or uncertain benefit**
TPO levels, bleeding time, serum complement

Abbreviation: CBC, Complete blood count; CMV, cytomegalovirus; EBV, Epstein–Barr virus; HBV, hepatitis B virus; HCV, hepatitis C virus; HIV, human immunodeficiency virus; PCR, polymerase chain reaction; TPO, thrombopoietin.

**Table 3:** Differential diagnosis of thrombocytopenia.

Bone marrow diseases, including myelodysplastic syndromes, leukemias, other neoplasms, metastatic disease, aplastic anemia, myelofibrosis, and Gaucher disease
Liver disease (including cirrhosis or portal hypertension)
Hereditary thrombocytopenia
Secondary immune thrombocytopenia, due to infections (HIV, HCV, HBV, *H. pylori*), autoimmune disorders/immunodeficiency (CVID, systemic lupus erythematosus, hyperthyroidism or APS), malignancy (for example, lymphoproliferative disorders)
Splenomegaly (Hypersplenism)
Drugs including heparin, alemtuzumab, PD-1 inhibitors, abciximab, valproate, alcohol abuse, quinine use, exposure to environmental toxins or chemotherapy, herbal products
Other microangiopathic disorders (DIC, TTP, HUS), Evans syndrome
Recent transfusions (post-transfusion purpura) and vaccinations
Others: vitamin B12 or folate deficiency, pregnancy, giant hemangiomas

Adapted from: Provan, D.; et al. *Blood Adv*. **2019**, *3 (22)*, 3780–3817 [85]. Abbreviation: APS, antiphospholipid syndrome; DIC, disseminated intravascular coagulation; CVID, common variable immunodeficiency; TTP, thrombotic thrombocytopenic purpura; HUS, hemolytic uremic syndrome; HBV, hepatitis B virus; HCV, hepatitis C virus; HIV, human immunodeficiency virus.

## Data Availability

Not applicable.
